# Supplementary figures and images for: The COVID-19 wave in Belgium during the Fall of 2020 and its association with higher education
Source: PLoS One. 2022 Feb 25;17(2):e0264516. doi: 10.1371/journal.pone.0264516 (PMC8880857; doi:10.1371/journal.pone.0264516)

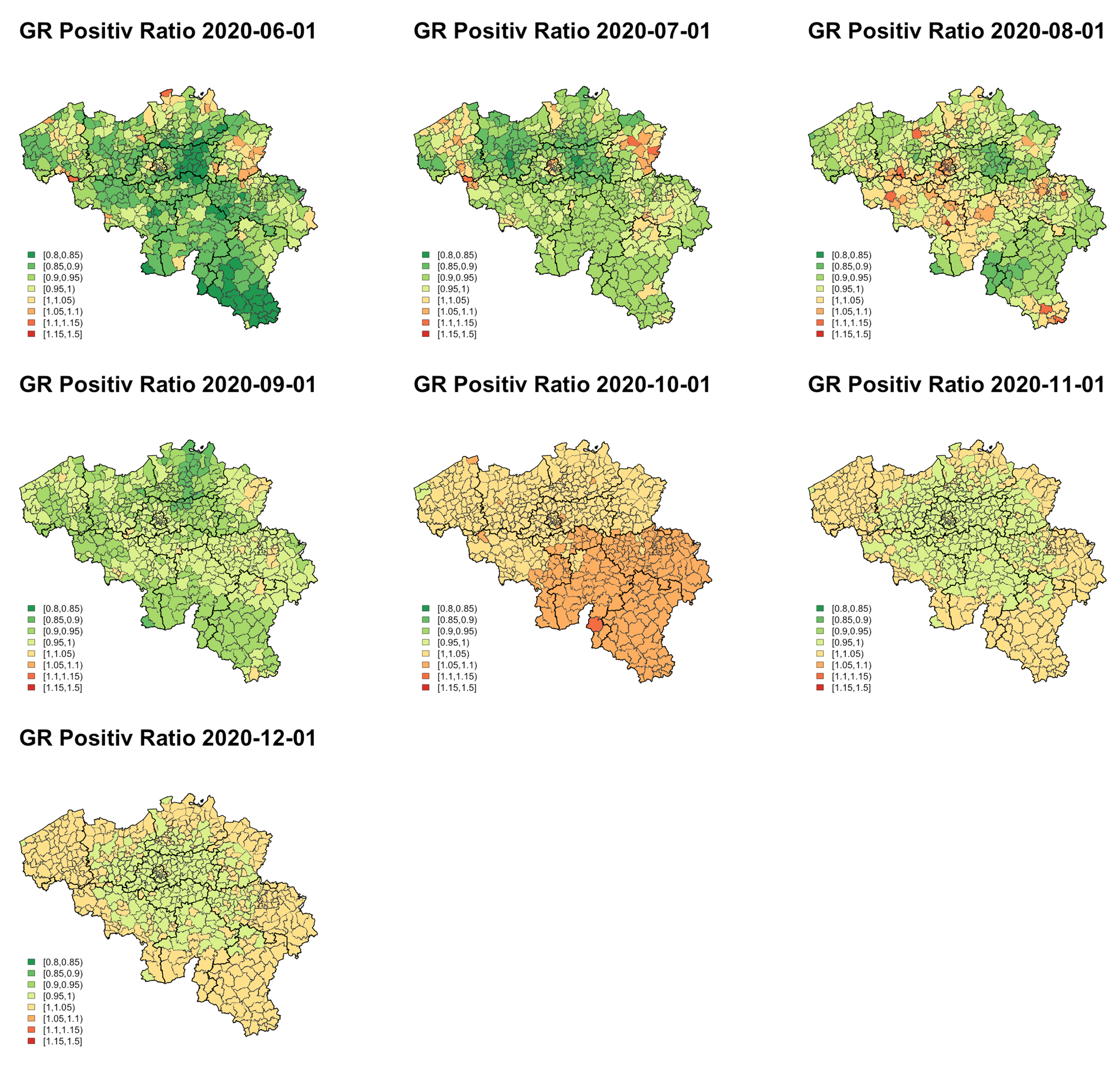

Supplement: S1 Fig — (TIF) [file pone.0264516.s001.tif]

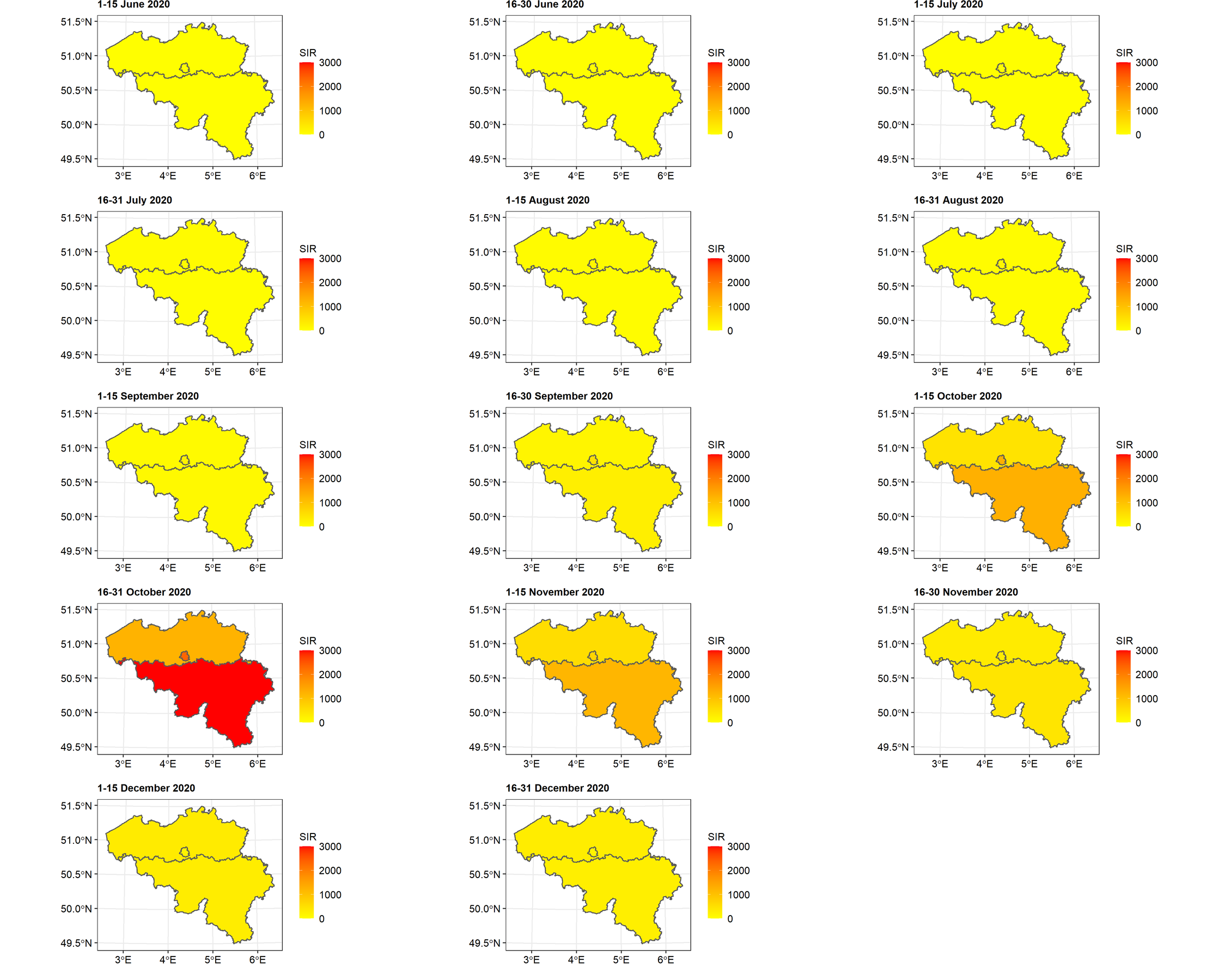

Supplement: S2 Fig — (TIF) [file pone.0264516.s002.tif]

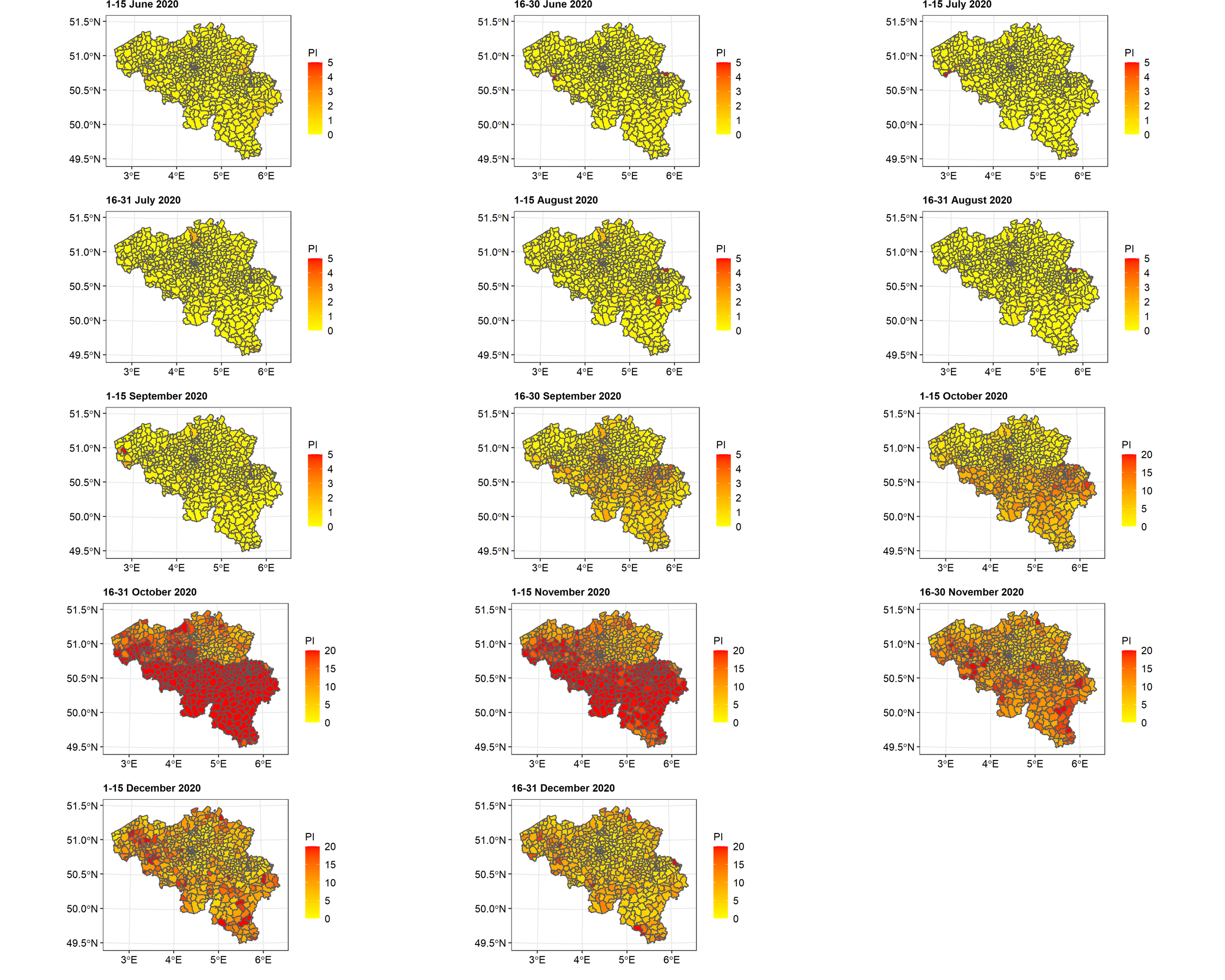

Supplement: S3 Fig — Different legend scales were used to accommodate the difference between two regions before and after September 2020. (TIF) [file pone.0264516.s003.tif]
