# Supplementary material for: The COVID-19 wave in Belgium during the Fall of 2020 and its association with higher education
Source: PLoS One. 2022 Feb 25;17(2):e0264516. doi: 10.1371/journal.pone.0264516 (PMC8880857; doi:10.1371/journal.pone.0264516)
Supplement: Attachment — Submitted filename: Response to Reviewers.pdf [file pone.0264516.s005.pdf]

# The COVID-19 wave in Belgium during the Fall of 2020 and its association with higher education

**Yessika Adelwin Natalia**[1,2] **Christel Faes**[1] **Thomas Neyens**[1,2]
**Geert Molenberghs**[1,2]

[1] *I-BioStat, Data Science Institute, Hasselt University, Hasselt, Belgium*
[2] *L-BioStat, Department of Public Health and Primary Care, Faculty of Medicine, KU Leuven, Leuven, Belgium*

Response to reviewers

First and foremost, we want to thank the reviewers for their critical assessment of our work and their constructive comments. We have addressed all of them and modified the paper accordingly. For your convenience, the reviewers' comments have been listed in order below in italics, followed by our responses in regular typeface.

## Academic editor

*Thank you for submitting your work to PLOS One. This is an important paper, which I feel could be further improved by addressing the comments below from myself and from the expert peer-reviewers.*

**Reply:** Thank you for your appreciation. All comments have been taken into account.

---

***Q AE.1*** *Please include the following items when submitting your revised manuscript: A marked-up copy of your manuscript that highlights changes made to the original version. You should upload this as a separate file labeled 'Revised Manuscript with Track Changes'.*

**Reply**: We have used Overleaf as LaTeX editor, therefore the track change feature is not available in the revised manuscript. As an alternative, we have used yellow highlighting to indicate the changes made.

---

***Q AE.2*** *Please ensure that your manuscript meets PLOS ONE's style requirements, including those for file naming.*

**Reply**: The manuscript is formatted using PLOS ONE LaTeX style formatting (Version 3.5 March 2018) found at https://journals.plos.org/plosone/s/file?id=SyJ3/plos-latex-template.zip. Authors affiliations and file naming are adjusted to meet the journal's requirements.

---

***Q AE.3*** *We note that you have indicated that data from this study are available upon request. PLOS only allows data to be available upon request if there are legal or ethical restrictions on sharing data publicly.*

**Reply**: The data are owned by a third-party organization (Sciensano) and cannot be made publicly available. Summarized versions are available in the open data repository, `https://epistat.wiv-isp.be/covid/`. To access the detailed data used in this study, a data request form found at `https://epistat.wiv-isp.be/datarequest/index.aspx` has to be returned to the Data Protection Officer office at Sciensano. To make this clear to the reader, we state in the manuscript:

> "All data used in this study were anonymous and therefore did not allow us to identify patients. The data are owned by Sciensano and cannot be made publicly available. Summarized versions are available in the open data repository, `https://epistat.wiv-isp.be/covid/`. To access detailed data and replicate our study findings, a data request form found at `https:`

//epistat.wiv-isp.be/datarequest/index.aspx has to be returned to the Data Protection Officer office at Sciensano."

---

***Q AE.4*** *Please include your full ethics statement in the 'Methods' section of your manuscript file. In your statement, please include the full name of the IRB or ethics committee who approved or waived your study, as well as whether or not you obtained informed written or verbal consent. If consent was waived for your study, please include this information in your statement as well.*

**Reply**: We included the full ethics statement in the 'Methods' (see also our response to the previous comment):

> "All data used in this study were anonymous and therefore did not allow us to identify patients. The data are owned by Sciensano and cannot be made publicly available. Summarized versions are available in the open data repository, https://epistat.wiv-isp.be/covid/. To access detailed data and replicate our study findings, a data request form found at https://epistat.wiv-isp.be/datarequest/index.aspx has to be returned to the Data Protection Officer office at Sciensano."

---

***Q AE.5*** *We note that Figures 2,4, 7, 8 S1-S3 in your submission contain [map/satellite] images which may be copyrighted.*

**Reply**: These figures were made using map images from a public domain website located at https://statbel.fgov.be/en/open-data. These open data are free to use for commercial as well as non-commercial purposes. The licence can be found at: https://statbel.fgov.be/sites/default/files/files/opendata/Licence%20open%20data_EN.pdf, which states that we only need to refer to the source and date of publication. We developed all images in this study with these publicly available data. We uploaded the permissions as an "Other" file with our submission.

---

***Q AE.6*** *The Introduction section is quite long and includes some information that might be better suited to the Discussions section.*

**Reply**: To accommodate this comment, we reorganized the 'Introduction' and moved some information to either 'Results' or 'Discussion'. This has led to considerable changes in the text. We invite the reviewer to consult these sections to gauge the changes.

---

***Q AE.7*** *Lines 21-23: In the beginning of the pandemic, sequencing was not routinely performed in all countries. Therefore, what was considered to be "a Spanish strain" might not have been Spanish in origin, but only undetected in countries that had not implemented sequencing yet. I would recommend revising this categorical statement that its spread "can only be explained by cross-border traveling." Same comment for lines 228-229.*

**Reply**: We entirely agree with this comment and thank the reviewer for raising this point. We adjusted this part to now read:

> "These authors have shown that a SARS-CoV-2 variant identified in Spain in early summer 2020, 20E (EU1), subsequently spread across Europe and this spread can only be explained by cross-border traveling."

and also

> "Of importance of Europe is the rapid spread of 20E (EU1) strain across the continent in the late summer of 2020."

***Q AE.8*** *Lines 33-35: Is there data to confirm or deny whether "The typical Belgian commuting customs" were maintained throughout the studied period in this paper? Many students in many countries changed their commuting customs either due to travel restrictions, or due to personal reasons, for example to avoid visiting elderly relatives or relatives with comorbidities.*

**Reply**: Travel restrictions within Belgium were not applied during our study period. Only in the Spring of 2020 was there a mandate to avoid non-essential travel. Therefore we assume that this typical commuting custom was maintained. Because some of the authors have served on official Belgian advisory boards (e.g., Geert Molenberghs for compulsory and higher education), we are quite confident that during the first few months of the academic year 2020-2021, the traditional commuting pattern stayed in place. Moreover, survey data corroborate this evidence. We have added some information to the second paragraph of the 'Introduction' as follows:

> "A small-scale survey conducted in a university in Belgium showed that COVID-19 did not influence the commuting behaviour of 47.5% of the students, 40.8% commuted less to home in the weekend, and 8,9% commuted more than before the pandemic [13]."

---

***Q AE.9*** *Lines 62-63: There is no need to include this statement, since all articles are structured this way: "This is followed by the results and conclusion from this study.".*

**Reply**: Thank you for this suggestion. We removed this part.

---

***Q AE.10*** *Some of the information presented in the Methods section might be a better fit in the Results section.*

**Reply**: Thank you for this suggestion. We moved some information from 'Methods' to 'Results'.

---

***Q AE.11*** *Please define the variable abbreviations in the logarithmic scale formula.*

**Reply**: We have revised the paragraph after the logarithmic formula to now read:

> "... with parameters $\alpha_{1k}$ and $\alpha_{2l}$ representing the means of age and gender groups. Municipality-specific covariates used in the model are: standardized mean income (as a proxy for the socioeconomic status of the municipality) denoted as $\texttt{income}_i$, logarithm of population density per km$^2$ denoted as $\texttt{log(popdens)}_i$, and standardized student ratio, i.e., the number of higher education students per 100 inhabitants in area $i$ denoted as $\texttt{sturatio}_i$. Note that we assume an age-specific parameter $\gamma_k$ for the student ratio, as interest is in the impact of higher education students on the incidence in each age group."

---

***Q AE.12*** *Was there an approval given by any Ethics committee or Institutional Review Board for this study? The authors only state that "All data used in this study were anonymous and therefore did not allow us to identify patients." However, ethics or institutional approvals still need to be obtained for the use of the data, at least from the data owner, even if direct identifiable patient data was not used in the study.*

**Reply**: We received the approval from data owner (Sciensano) as we mentioned in our response to Q AE.3. Given that the data were anonymous, no further institutional ethics review was necessary. Additional explanation is added to our 'Methods' section.

---

***Q AE.13*** *Line 203-205: Please specify during which period in the pandemic.*

**Reply**: We added the following sentence:

> "Some studies, conducted during the initial phase of the COVID-19 pandemic (March–April 2020), show that younger adults were more likely to leave their homes frequently [36] and take part in social activities such as parties, fitness, or casual hang-outs [37–40]."

***Q AE.14*** *Is there data regarding the predicted incidence vs. the actual incidence during the study time span?*

**Reply**: In our analysis, we used the actual incidence as input and then refined it with other covariates to obtain model-based predictions. Our main goal is to explore the effect of these covariates. In our opinion, a validation of predicted incidences is not really central to the question under investigation in this manuscript. Therefore, the reader can get an appreciation of actual versus predicted incidence in Figures 2 and 6.

***Q AE.15*** *Since the study data was collected prior to the widescale deployment of COVID vaccination, the results can only be representative for an unvaccinated population. Since the age groups analyzed here are now included among those eligible to receive vaccination in most countries, this would be expected to significantly change the landscape for the upcoming start of the new education year. This should be discussed as a study limitation and further information should be provided regarding the vaccination program in Belgium, i.e., which age groups are now eligible, with particular emphasis on the student population, discussed in parallel with the vaccine uptake in this particular population group.*

**Reply**: We appreciate this comment and have added the following wording to the 'Discussion':

> "Last but not least, this study was conducted prior to the wide-scale deployment of COVID-19 vaccination. Therefore, the results cannot be interpreted as directly representative for a vaccinated population. The Belgian government started the vaccination campaign in late December 2020, with a primary focus on nursing home residents, healthcare workers, patients with comorbidities, and then the general population in decreasing age bands. In late August 2021, a bit over 70% of the Belgian population is fully vaccinated with adult coverage (18+) of 83% [50]. Vaccination is one of the primary mitigation strategies to avoid future higher-education induced COVID-19 waves. Even though current vaccination coverage is satisfactory, [51] reported that levels of willingness to accept a COVID-19 vaccine in many countries are insufficient to meet the requirements for herd immunity or even for a vaccination level that obviates the more stringent non-pharmaceutical interventions, underscoring the importance of the latter, particularly when transmission of COVID-19 is still relatively high in some populations."

# Reviewer 1

*The authors' efforts at demonstrating that, policies such as opening of higher education during an ongoing pandemic has consequences on the evolution of the pandemic is worth praising. However, the study is that of an ecological study design and the authors failed to convincingly show the association being described. Below are some of my concerns.*

**Reply:** We thank the reviewer for this appraisal of our paper. The below comments have been taken into account. Point-by-point responses are provided.

***Q 1.1*** *Generally, all the figures were difficult to understand due to poor readability of legends and quality of figures making understanding of the figures a problem.*

**Reply**: Thank for for noting this limitation in the original manuscript. We adjusted the figures with higher resolution in our revised manuscript.

***Q 1.2*** *Also, some figures lack the necessary description of axis eg; Fig 1, x-axis is missing.*

**Reply**: We adjusted this figure to include the $x$-axis label.

***Q 1.3*** *Difficult to understand the message from the map.*

**Reply**: It can indeed have been difficult to evaluate the main message presented by the map in its previous resolution. We have adjusted the figures to clarify this. Our aim is to show the contrast between different

periods and municipalities in Belgium in terms of COVID-19 incidence rates. Higher incidence rates could be observed in the beginning of October 2020, particularly in the Walloon and Brussels regions. In some municipalities, the incidence rates continue to be high even when the overall incidence rate declined.

---

***Q 1.4*** *Figure one was referenced in the introduction, second paragraph; I was not sure even after looking at figure one, if it is as a result of your analysis or copied from elsewhere. If copied, then the source of the fig, copyright issues need to be described. If it is as a result of your own analysis, then I would consider putting it in the results section and not introduction. Similarly, Figure two was placed in the introduction and discussed. I would have considered all discussion in the appropriate column.*

**Reply**: We appreciate this comment and moved this information to the 'Results' section since this is based on our own analysis. It automatically implies that there are no copyright issues.

---

***Q 1.5*** *Method section, paragraph 3, line 82 stated that "All data are obtained from Statbel, for the year 2016." However, checking the open data section of statbel, it was obvious that it has up to date data to even 2021. The "Population by place of residence, nationality, marital status, age and sex" for 2020 is available. What informed the choice of 2016 dataset when 2020 dataset is available for direct comparison with 2020 COVID-19 data.*

**Reply**: Thank you for pointing this out. We used population data of year 2020. However, contrary to many other characteristics, the last census on the student population was conducted in 2016. Therefore, we decided to use number of students and mean income per municipality from the year 2016. Note that the student population is relatively stable over time. We rephrased this part as follows:

> "We obtained population data for the year 2020. However, the last census count of the student population was conducted in 2016, which we used to calculate the number of students and the mean income per municipality."

---

***Q 1.6*** *Line 83 mentioned that, "The student ratio is presented in the upper right panel" of figure 4. However, this is not obvious from the figure 4.*

**Reply**: To accommodate this comment, we adjusted Fig. 4 (which became Fig. 3) and use letters (a–d) to refer to the correct figure.

---

***Q 1.7*** *Results section; line 134-139 stated that, "We observe that the age group of 18–29 years, followed by age group 30–39 years had the highest predicted incidence of COVID-19 cases, particularly from mid-September 2020 until the end of October 2020, in both Flanders and Wallo-Brux. From November 2020 onwards, the predicted incidence was highest within the group of those older than 80 years in both regions. This suggests that this epidemic wave started in the younger individuals, and that infection spread from the younger individuals to the elderly population." a. In as much as your assertion may be possible, there are other factors eg; relatively severe disease in the 80-year-old group from November onwards such that, the older age group are presenting for testing and the relatively asymptomatic/ mildly symptomatic younger age group are not presenting for testing. b. Also are you aware what variant of the virus was in circulation around November onwards, which may be different from that which was in circulation around September. The differences in the transmission and characteristics of the variants in circulation could also explain the observation.*

**Reply**: We agree that the younger age groups are less likely to present for testing. But precisely because of this, the observation that the younger age groups have higher incidences in the early period (September and October 2020) underscores that the circulation largely initiated in this age group. Contact data studies underscore this. Yet, in the later period (November 2020) this is reversed and older age groups have an increased share of confirmed cases. Throughout the period under investigation, there are no variants of concern circulation, other than wildtype (cf. the spread of 20E (EU1)). We added to the paper:

> "Indeed, given that younger age groups are less likely to present for testing, but given that nevertheless they are very prominently present among confirmed cases in September-October 2020, underscores this point. We also note that there is no evidence for variants-of-concern circulating over the study period. The alpha variant was identified in Belgium in December 2020 for the first time."

---

***Q 1.8*** *We need to understand how COVID-19 cases are tested in Belgium. Is it the situation as in many countries that symptomatic or people meeting a set of case definitions are tested.*

**Reply**: Over a long period of time, encompassing the study period, COVID-19 testing has targeted possible COVID-19 cases (symptoms), high-risk contacts (e.g., as identified via contact tracing), travelers, patients prior to receiving medical care, etc. An important exception is the period from late October 2020 to late November 2020 during which, because of very high circulation (up to 20,000 confirmed cases per day), asymptomatic cases were no longer tested. We included this information in the sixth paragraph of the 'Discussion' (see Q 1.13) and summarized the test strategies in Figure 5.

---

***Q 1.9*** *The figures and graphs did not illustrate the association of increased incidence and the reopening of schools convincingly. It could also be due to the fact that the legends of the graphs are not legible, however, I was expecting a basic epi curve showing the time line of reopening of higher education and other possible interventions.*

**Reply**: Thank your for this comment. To offer a better perspective regarding increased incidence and the reopening of higher education, we have added the epidemiological curve of COVID-19 daily cases, which also features preventive measures applied during our study period (Fig. 5).

---

***Q 1.10*** *Conclusion, lines 242-244 stated that "The results in this study provide insights into the association between reopening higher education in the academic year 2020-2021 and increased COVID-19 incidence in the Fall wave. However, this conclusion is not obvious from the graphs given.*

**Reply**: We adjusted the quality of the graphs and added extra information as mentioned in Q 1.9.

---

***Q 1.11*** *Also, your study only addressed higher education. What about other schools which were non-higher education? Could the reopening of that also affect the findings?*

**Reply**: We appreciate the comment about compulsory education (in particular primary and secondary education), as a phenomenon to be studied relative to higher education. However, there are few reasons to keep this outside of the scope of the current paper. The most important one is that incidences in the compulsory school population, monitored directly by the school system itself have been considerably lower than that of the general population, from the start of the school year (September 1, 2020) until February 2021. For example, on November 1, 2021, around the peak of the second wave, the incidence in the Flemish population was 1118, while that in the compulsory school system, among pupils, 355. Compulsory education is also more homogeneously spread throughout the population, precisely because of its compulsory nature. We have added to the paper:

> "(. . . ), we focus on higher education alone, rather than on compulsory education as well. There are two more arguments to justify this. First, incidences in the compulsory school population, monitored directly by the school system itself have been considerably lower than that of the general population, from the start of the school year (September 1, 2020) until February 2021. For example, on November 1, 2021, around the peak of the second wave, the incidence in the Flemish population was 1118, while that in the compulsory school system, among pupils, 355. Second, compulsory education is also more homogeneously spread throughout the population (both in terms of school location as geographical spread of pupils), precisely because of its compulsory nature."

***Q 1.12*** *The age group which was supposed to be in higher education as described 18-29, 30-39 also constitute the working age group. In most countries, reopening of higher education occurred with relaxing of restriction allowing the working group to engage in almost all possible economic activities. It is not convincing from your findings if the increase in incidence was mainly due to reopening of higher education.*

**Reply**: We agree that these age groups also encompass the working age group. We added a discussion on this topic to the third paragraph of 'Discussion' as follows:

> "Clearly, higher-education students belong to an age group that partly consists of working individuals [42], which a priori might complicate understanding dynamics in COVID-19 transmission in this age group, particularly after reopening of higher education. However, the most recent relaxations, taking place prior to the start of the academic year, did not address the workplace and teleworking had been recommended uninterruptedly since July 2020; further relaxation in economic activities did not occur until December 2020 (Fig. 5). Based on this, we expect that the impact of behavior in the working sub-population within this age group on changes in COVID-19 incidence rates is minimal."

***Q 1.13*** *Your study design is that of an ecological study, which comes with some limitations and biases. a. Patient consultation habits, screening methods across the geographical regions may have an effect on the findings which need explanation b. Diagnostic criteria over time and across geographical region could also play a part in the observed differences which needs explanation on how such might have been addressed or minimized.*

**Reply**: Thank you for noting these limitations. To accommodate this comment, we adjusted our sixth paragraph in the 'Discussion' Section to now read:

> "Fourth, there are spatio-temporal dynamics in COVID-19 testing strategies and test capacity [48]. We therefore analyzed the data per region, since these dynamics were mainly driven by COVID-19 preventive measures, a considerable part of which was based on decisions taken by regional authorities. Over a long period of time, encompassing the study period, COVID-19 testing has targeted possible COVID-19 cases (symptoms), high-risk contacts (e.g., as identified via contact tracing), travelers, patients prior to receiving medical care, etc. An important exception is the period from late October 2020 to late November 2020 during which, because of very high circulation (up to 20,000 confirmed cases per day), asymptomatic cases were no longer tested. We found a clear north-south divide in the growth rate (Fig. 4) as well as the positivity ratio (S1 Fig). Sciensano reported that the number of COVID-19 tests performed in the Flemish Region was 22.9% higher when compared to the Walloon and Brussels in this period [49]. There is a possibility that many asymptomatic or mild cases could not be detected in Wallo-Brux Region. Evidently, without proper quarantine procedures, the infected person could further transmit the virus in the population."

# Reviewer 2

*Although the authors have done this study using COVID-19 data only for Belgium, this is an expedient work for the whole world during the ongoing pandemic situation. This study could help decision makers to make appropriate decision to lessen the burden of COVID-19. I would like to recommend this manuscript for possible publication after addressing the following minor issues:*

**Reply:** We thank the reviewer for these very positive comments. All suggestions have been taken into account.

***Q 2.1*** *The horizontal axis-label should be added in Fig 1.*

**Reply**: We adjusted Fig. 1 to include the horizontal axis label.

---

***Q 2.2*** *The figure quality should be increased.*

**Reply**: We agree with this comment and we have adjusted the figures towards higher resolution.

---

***Q 2.3*** *The legend of all figures should be clearly visualized.*

**Reply**: We have adjusted the legends so that they could be clearly seen.

---

# Reviewer 3

*Article is well written with scientific explanation but its more focusing on the data associated with higher association but not gave explanation about opening of middle and lower school, inclusion or comparison with more data may give more accuracy to study.*

**Reply:** We are grateful for the reviewer's nice appraisal of our work. We appreciate the comment about compulsory education (in particular primary and secondary education), as a phenomenon to be studied relative to higher education. However, there are few reasons to keep this outside of the scope of the current paper. The most important one is that incidences in the compulsory school population, monitored directly by the school system itself have been considerably lower than that of the general population, from the start of the school year (September 1, 2020) until February 2021. For example, on November 1, 2021, around the peak of the second wave, the incidence in the Flemish population was 1118, while that in the compulsory school system, among pupils, 355. Compulsory education is also more homogeneously spread throughout the population, precisely because of its compulsory nature. We have added to the paper:

> "(. . . ), we focus on higher education alone, rather than on compulsory education as well. There are two more arguments to justify this. First, incidences in the compulsory school population, monitored directly by the school system itself have been considerably lower than that of the general population, from the start of the school year (September 1, 2020) until February 2021. For example, on November 1, 2021, around the peak of the second wave, the incidence in the Flemish population was 1118, while that in the compulsory school system, among pupils, 355. Second, compulsory education is also more homogeneously spread throughout the population (both in terms of school location as geographical spread of pupils), precisely because of its compulsory nature."

---

# Reviewer 4

*The manuscript is quite an interesting read and enjoyable! The analysis and results were presented well, as well as noted the limitations of the methods. The figures, however, are quite hard to read and needs to be provided in a higher resolution. I wasn't able to scrutinize the results because of that, so it will be good to have the figures re-sent.*

**Reply:** We thank the reviewer for these very nice comments. We agree that the figures are quite hard to read. Therefore we adjusted the figures to higher resolution in our revised manuscript.
