# Supplementary material for: The COVID-19 wave in Belgium during the Fall of 2020 and its association with higher education
Source: PLoS One. 2022 Feb 25;17(2):e0264516. doi: 10.1371/journal.pone.0264516 (PMC8880857; doi:10.1371/journal.pone.0264516)
Supplement: Attachment — Submitted filename: Response to Reviewers.pdf [file pone.0264516.s006.pdf]

The COVID-19 wave in Belgium during the Fall of 2020 and its association with higher education

Yessika Adelwin Natalia[1] **Christel Faes**[1] **Thomas Neyens**[1,2]
Geert Molenberghs[1,2]

[1] *I-BioStat, Data Science Institute, Hasselt University, Hasselt, Belgium*
[2] *L-BioStat, Department of Public Health and Primary Care, Faculty of Medicine, KU Leuven, Leuven, Belgium*

Response to reviewers

We want to thank again the reviewers for their critical assessment of our previous manuscript and their constructive comments. We have addressed all of them and modified the paper accordingly.

Academic editor

Q AE.1 *In the newly added phrase on line 23, please correct the decimal indicator to a dot instead of a comma.*

Reply: We have corrected the decimal indicator.

Q AE.2 *In the newly added phrase: "First, incidences in the compulsory school population, monitored directly by the school system itself have been considerably lower than that of the general population, from the start of the school year (September 1, 2020) until February 2021." – This might no longer be the case with surges of cases among school children in many European countries this autumn, now that delta has become the dominant variant. This could be briefly addressed as part of the discussion.*

Reply: Thank you for this comment. We added a brief discussion on this topic to the last paragraph of the 'Discussion' as follows:

> "... During Fall wave of 2020, which was analysed here, COVID-19 incidence in the compulsory school population was considerably lower than in the general population, while this was not the case for the Fall wave of 2021, when incidence in children aged 0–9 years old was reported to be 5.3 times higher than during the same period in 2020. For adults between 20–49 years old, the COVID-19 incidence was increased around 1.04 times [54]. ..."

Q AE.3 *In the newly added phrase "Some studies, conducted during the initial phase of the COVID-19 pandemic (March–April 2020), show that younger adults were more likely to leave their homes frequently [36] and take part in social activities such as parties, fitness, or casual hang-outs [37-40]" one further aspect could be considered, i.e., that younger children were generally "shielded" during the initial phase of the pandemic by their parents who continued to engage in work-related and social activities [see ref PMID 33489943]. It would be interesting to see how and if this translates into "shielding" of older children, i.e., university students engaged in higher education. This could be briefly addressed as part of the discussion.*

Reply: ver 2 : Thank you for this comment. We briefly addressed this topic in lines 241–244 as follows:

> "... Note that we expect this to be less the case for children. Moreover, it has been documented that parents during the pandemic's first wave often protected their children against important transmission routes, even when they would continue to have work-related and social contacts [42]. ..."

Q AE.4 *Please add a brief comment to the discussion section to address the change in transmission dynamics with the delta variant and to discuss how your results could be transposable or not to the new transmission scenario in areas where delta is now the main circulating variant.*

Reply: We are grateful for this comment. We added a brief discussion on this topic to the last paragraph of 'Discussion' as follows:

> "The dynamics of COVID-19 transmission changes rapidly and at the time of writing (December 2021), the Delta followed by the Omicron variants are the main circulating variants globally [6]. In Belgium, more than 90% of the infection is caused by the Delta variant since late July until early December 2021. Consequently, COVID-19 incidence in Belgium is also increased considerably due to the increased transmissibility of this variant. During Fall wave of 2020, which was analysed here, COVID-19 incidence in the compulsory school population was considerably lower than in the general population, while this was not the case for the Fall wave of 2021, when incidence in children aged 0–9 years old was reported to be 5.3 times higher than during the same period in 2020. For adults between 20–49 years old, the COVID-19 incidence was increased around 1.04 times [54]. There are many factors that contribute to the virus transmission. However, we observed similar trend in year 2020 and 2021, i.e. a considerable increase of COVID-19 incidence after the beginning of the new academic year, despite the shift of transmission dynamics in younger children. This underscores the relevance of our results in the new transmission scenario."
